# Age-associated repression of type 1 inositol 1, 4, 5-triphosphate receptor impairs muscle regeneration

**DOI:** 10.18632/aging.101039

**Published:** 2016-09-21

**Authors:** Jeong Yi Choi, Chae Young Hwang, Bora Lee, Seung-Min Lee, Young Jae Bahn, Kwang-Pyo Lee, Moonkyung Kang, Yeon-Soo Kim, Sun-Hee Woo, Jae-Young Lim, Eunhee Kim, Ki-Sun Kwon

**Affiliations:** ^1^ Aging Research Center, Korea Research Institute of Bioscience and Biotechnology (KRIBB), Daejeon 34141, Republic of Korea; ^2^ College of Biological Science and Biotechnology, Chungnam National University, Daejeon 34134, Republic of Korea; ^3^ Department of Bio and Brain Engineering, Korea Advanced Institute of Science and Technology, Daejeon 34141, Republic of Korea; ^4^ Department of Functional Genomics, Korea University of Science and Technology, Daejeon 34113, Republic of Korea; ^5^ Graduate School of New Drug Discovery & Development, Chungnam National University, Daejeon 34143, Republic of Korea; ^6^ College of Pharmacy, Chungnam National University, Daejeon 34143, Republic of Korea; ^7^ Department of Rehabilitation Medicine, Seoul National University Bundang Hospital, Gyeonggi-do 13620, Republic of Korea

**Keywords:** ITPR1, muscle aging, muscle regeneration, sarcopenia, U0126

## Abstract

Skeletal muscle mass and power decrease with age, leading to impairment of mobility and metabolism in the elderly. Ca^2+^ signaling is crucial for myoblast differentiation as well as muscle contraction through activation of transcription factors and Ca^2+^-dependent kinases and phosphatases. Ca^2+^ channels, such as dihydropyridine receptor (DHPR), two-pore channel (TPC) and inositol 1,4,5-triphosphate receptor (ITPR), function to maintain Ca^2+^ homeostasis in myoblasts. Here, we observed a significant decrease in expression of type 1 IP3 receptor (ITPR1), but not types 2 and 3, in aged mice skeletal muscle and isolated myoblasts, compared with those of young mice. ITPR1 knockdown using shRNA-expressing viruses in C2C12 myoblasts and tibialis anterior muscle of mice inhibited myotube formation and muscle regeneration after injury, respectively, a typical phenotype of aged muscle. This aging phenotype was associated with repression of muscle-specific genes and activation of the epidermal growth factor receptor (EGFR)-Ras-extracellular signal-regulated kinase (ERK) pathway. ERK inhibition by U0126 not only induced recovery of myotube formation in old myoblasts but also facilitated muscle regeneration after injury in aged muscle. The conserved decline in ITPR1 expression in aged human skeletal muscle suggests utility as a potential therapeutic target for sarcopenia, which can be treated using ERK inhibition strategies.

## INTRODUCTION

Sarcopenia, age-related loss of muscle quantity and quality, is a crucial determinant of geriatric fragility [1]. Sarcopenia increases susceptibility to muscle damage, serious falls, obesity and diabetes [2]. Age-related changes in muscle are thought to depend on a decrease in muscle stem cells and their niche [3, 4], which results in global changes in associated gene and protein expression [5-7] as well as posttranslational modifications [8].

Skeletal muscle regeneration is a multistep process [9]. In response to stimuli generated by exercise or injury, satellite cells re-enter the cell cycle to produce myoblasts, subsequently withdraw from the cell cycle, and differentiate into myocytes, which fuse into new myotubes or with host myofibers. This fusion process is crucial for postnatal growth, maintenance and repair of skeletal muscle in the adult stage. Myotube formation is completely Ca^2+^ dependent, and requires net Ca^2+^ influx into myoblasts [10–12]. Ca^2+^ -dependent enzymes, such as Ca^2+^/calmodulin-dependent protein kinase (CaMK) and calcineurin, are required for activation of the muscle-specific transcription factors myogenin [13] and myocyte enhancer factor 2 (MEF2) [14,15]. Stromal interaction molecules (STIM) 1 and 2 (endoplasmic reticulum Ca^2+^ sensors) and the Orai1 Ca^2+^ channel involved in store-operated Ca^2+^ entry (SOCE) are crucial for hyperpolarization and subsequent induction of myoblast differentiation [16]. Transient receptor potential canonical (TRPC) 1 and 4 that also participate in SOCE are essential for myoblast fusion [17]. Inositol 1,4,5-triphosphate receptors (ITPR), Ca^2+^ release channels activated by inositol 1,4,5-triphosphate (IP3) that are required for endogenous spontaneous Ca^2+^ transients and SOCE, are also essential for the early steps of myoblast differentiation [18].

With aging, skeletal muscle shows impaired myogenic potential, which, in turn, induces atrophy [19,20]. Ca^2+^ signaling molecules are reported to be associated with age-dependent muscle degeneration. In aged muscle, decreased expression of mitsugumin-29 induces abnormal interaction of dihydropyridine receptor (DHPR) with ryanodine receptor 1 (RyR1), which leads to compromised Ca^2+^ spark signaling [21]. RyR1 from aged mice is oxidized and cysteine-nitrosylated, resulting in leaky channels with increased open probability, which causes muscle weakness [8]. However, RyR1 is not expressed by undifferentiated myoblasts and does not affect the myogenic potential of these cells [22]. Therefore, alternative mechanisms may underlie the abnormal regulation of intracellular Ca^2+^ in impaired differentiation of aged myoblasts.

Among the various Ca^2+^ sensors and channels, ITPR1 expression was dramatically decreased in aged muscles and myoblasts. We demonstrated that ITPR1 modulates intracellular calcium oscillation that plays a crucial role in skeletal muscle regeneration. Our data suggest that loss of ITPR1 with age is one of the major underlying causes of sarcopenia.

## RESULTS

### ITPR1 levels decline in aged muscle

**Figure 1:**
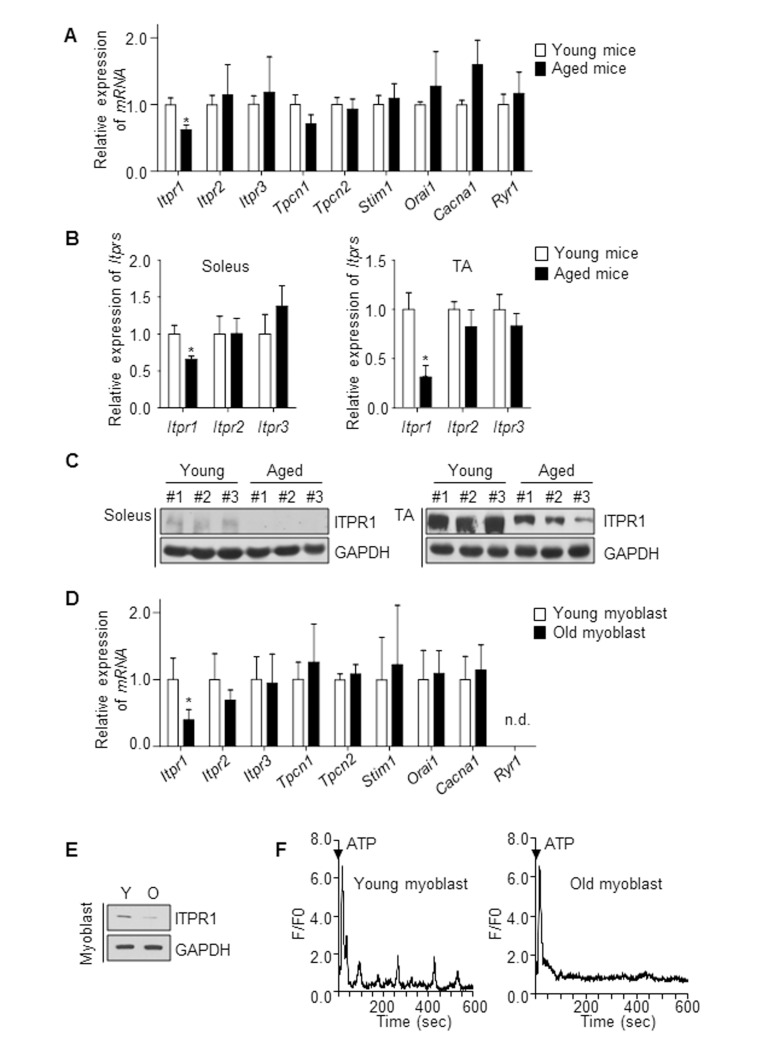
ITPR1 levels are decreased in aged skeletal muscle (**A**) qRT-PCR analysis of mRNA levels of Ca^2+^ regulatory genes, relative to *36b4*, in young (6 months) and aged (28 months) mice gastrocnemius muscle. *n* = 5 for each group. (**B**) qRT-PCR analysis of *Itpr* mRNA levels, relative to *36b4*, from soleus and TA muscles of young (isolated from 6 month-old mice) and aged (isolated from 28 month-old mice) mice. *n* = 5 for each group. (**C**) Immunoblot analysis of ITPR1 protein levels from soleus and TA muscles of young and aged mice, with GAPDH as the loading control. *n* = 3 for each group. (**D**) qRT-PCR analysis of mRNA levels of Ca^2+^ regulatory genes, relative to *36b4*, in young (isolated from 3 month-old mice) and old (isolated from 28 month-old mice) primary myoblasts. *n* = 5 for each group. (**E**) Immunoblot analysis of ITPR1 protein levels in young and old primary myoblasts, with GAPDH as the loading control. (**F**) Measurement of extracellular ATP (1 mM)-induced Ca^2+^ oscillations in a single myoblast from young and old primary myoblast cultures. *F/F0* represents the change in fluorescence normalized to resting fluorescence (*F0*).

Aged muscle displays defects in regeneration. Ca^2+^ is a universal intracellular messenger that modulates many aspects of muscle physiology, such as myotube formation [10–12], muscle contraction and mechanical strength [8, 21]. To examine the changes in expression patterns of Ca^2+^ modulators with aging, we isolated the gastrocnemius muscle of young (6 months) and aged (28 months) mice, and analyzed the expression profiles of several Ca^2+^ channels and sensors, including *Itprs* (ITPR), *Tpcn1* (two-pore channel, TPC1), *Tpcn2* (TPC2), *Stim1* (STIM1), *Orai1* (Orai1), *Cacna1* (DHPR) and *Ryr1* (RyR1). Among these, *Itpr1* mRNA expression in gastrocnemius muscle decreased by ∼40% with age while other Ca^2+^ channels and sensors levels remained unchanged (Fig. 1A). We further isolated two types of hindlimb muscle, soleus (slow-twitch) and tibialis anterior (TA, fast-twitch), in both young and aged mice, and analyzed the mRNA and protein levels of ITPRs (Fig. 1B, C). Both mRNA and protein levels of ITPR1 declined with age (Fig. 1B-C) while those of the ITPR2 and ITPR3 isoforms and other Ca^2+^ channels remained unchanged (Fig. 1B). Primary myoblasts were isolated from hindlimb muscles of 3 month (hereafter designated “young myoblasts”) and 28 month-old mice (hereafter designated “old myoblasts”) [23], and the mRNA expression profiles of *Itprs*, *Tpcn1*, *Tpcn2, Stim1, Orai1, Cacna1* and *Ryr1* evaluated. Notably, Itpr1 mRNA expression in myoblasts decreased by ∼60% in old myoblasts while Itpr2 and Itpr3 levels remained unchanged (Fig. 1D). The ITPR1 protein level was similarly decreased in old myoblasts (Fig. 1E). Next, using human biopsy samples of six young (27 to 55 years old) and six aged (66 to 79 years old) subjects, we compared the ITPR1 levels in the vastus lateralis (see below in Fig. 4B). Consistently, human skeletal muscle of the aged group exhibited decreased ITPR1 expression.

To determine the cellular consequence of this decline in ITPR1, Ca^2+^ oscillation was monitored in old myoblasts relative to young myoblasts. Intracellular Ca^2+^ oscillation induced by ATP stimulation [24] was almost undetectable in old primary myoblasts, compared to that in young myoblasts (Fig. 1F). Ca^2+^ signals, in particular, Ca^2+^ oscillations, are known to play a crucial role in modulating gene expression in several systems, including T lymphocytes [25], neurons [26] and muscle cells [18]. Based on the collective results, we propose that the decreased levels of ITPR1 and suppression of associated Ca^2+^ signaling are important contributory factors to muscle aging.

### Role of ITPR1 in myogenic differentiation

Old myoblasts show decreased capability of fusion into myotubes under differentiation conditions, compared to young myoblasts [27] (Supplementary Fig. S1).

Myotube formation is strictly Ca^2+^-dependent, and inhibited by depletion of Ca^2+^ stores [10]. However, the issue of whether Ca^2+^ channels or sensors contribute to the dysregulation of myotube formation with aging remains to be established.

**Figure 2:**
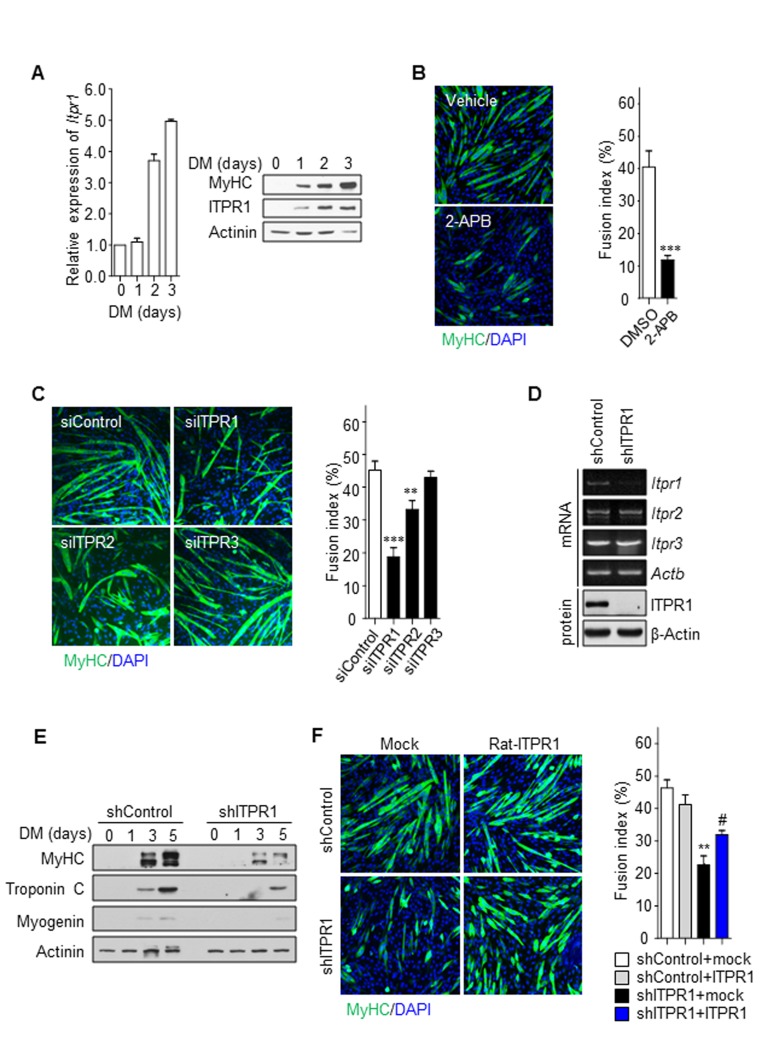
Inhibition of ITPR1 impairs myotube formation (**A**) C2C12 myoblasts were induced to differentiate for 3 days and harvested at the indicated time-points. qRT-PCR and immunoblot analysis of mRNA and protein levels for ITPR1 in differentiating C2C12 cells. Actinin was used as the loading control. (**B**) C2C12 myoblasts were induced to differentiate for 5 days in the presence of 2-APB or vehicle and stained with MyHC (green) antibody and DAPI (blue). (**C**) C2C12 myoblasts were transfected with siRNA specific for each ITPR isoform or scrambled siRNA, induced to differentiate for 5 days, and stained with MyHC (green) antibody and DAPI (blue). (**D**) RT-PCR and immunoblot analyses of mRNA and protein levels of the three ITPR isoforms, respectively, in ITPR1-silenced C2C12 myoblasts. β-Actin was used as the loading control. (**E**) C2C12 myoblasts were transfected with siRNA against ITPR1 or scrambled siRNA. Cells were induced to differentiate for 5 days, harvested at the indicated time-points, and analyzed via immunoblotting with antibodies against MyHC, Troponin C and Myogenin. Actinin was used as the loading control. (**F**) Stable C2C12 cell lines expressing shRNA against ITPR1 or scrambled shRNA were transiently transfected with rat ITPR1 expression plasmids and induced to differentiate for 5 days, followed by staining with MyHC (green) antibody and DAPI (blue). The fusion index was calculated as the ratio of the number of multinucleated MyHC-positive myotubes to the number of total cells counted based on DAPI staining.

To determine if ITPR1 is responsible for impaired myotube formation with aging, we followed their expression level during differentiation using quantitative real-time PCR and immunoblot analysis (Fig. 2A). We found that ITPR1 mRNA and protein expression levels were gradually increased after differentiation. This result imply that ITPR1 may play an important role in myotube formation. To evaluate a putative role of ITPR1 on muscle homeostasis, we examined whether loss of ITPR1 alters myogenic differentiation in the C2C12 myoblast cell. Interestingly, treatment with 2-aminoethoxydiphenyl borate (2-APB), a specific inhibitor of ITPR, led to deceleration of C2C12 myoblast differentiation (Fig. 2B). Transient knockdown of ITPR1, ITPR2 and ITPR3 in C2C12 myoblasts was further performed, and myogenesis examined relative to control siRNA-transfected C2C12 myoblasts. Among the three isoforms, cells with ITPR1 knockdown showed severe defects in myotube formation whereas ITPR2 and ITPR3 knockdown had non-significant effects (Fig. 2C). A stable cell line with decreased ITPR1 expression established from C2C12 myoblasts infected with shRNA-expressing lentivirus (Fig. 2D) similarly exhibited a marked reduction in myotube formation along with decreased expression of myosin heavy chain (MyHC), Troponin C and Myogenin upon exposure to differentiation conditions (Fig. 2E and Supplementary Fig. S2A). These phenotypes of ITPR1 knockdown C2C12 myoblasts were effectively rescued by ectopic expression of ITPR1 (Fig. 2F, Supplementary Fig. S2B and C). ITPR1-depleted myoblasts showed impaired Ca^2+^ oscillations similar to those observed for old myoblasts, with no changes in the resting Ca^2+^ content (Supplementary Fig. S3). Our data suggest that decreased expression of ITPR1 in old myoblasts leads to defective myogenesis.

### ITPR1 knockdown induces C2C12 myoblast proliferation via the EGFR-Ras-ERK signaling pathway

Quiescent satellite cells are activated to form myogenic precursor cells, which undergo multiple rounds of division prior to terminal differentiation and fusion to form multinucleated myofibers. Activated satellite cells also proliferate to generate progeny that restore the pool of quiescent satellite cells [28]. This pivotal mechanism of proliferation and differentiation in satellite cells is conserved in both primary and C2C12 myoblast cultures [29, 30].

**Figure 3:**
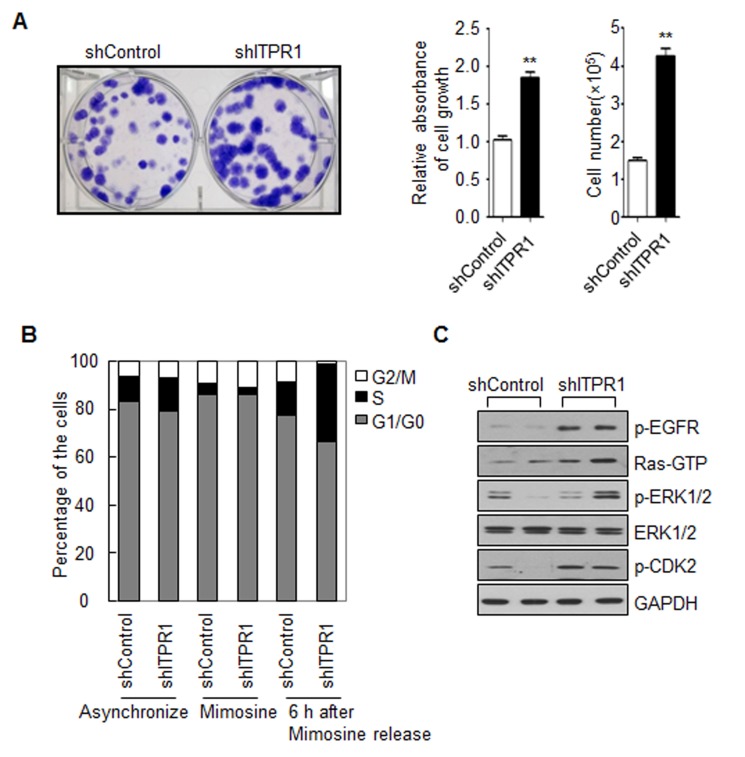
ITPR1 knockdown increases C2C12 cell proliferation through activation of the EGFR-Ras-ERK pathway (**A**) ITPR1 knockdown and control C2C12 myoblasts were seeded at a density of 1×10^2^ cells per well in 6-well plates. Cells were allowed to grow for 10 days until small colonies were clearly formed. Colonies were stained with crystal violet solution for 1 h. Cell-bound dye was eluted with a 1:1 solution of 0.1 M sodium citrate, pH 4.2, and ethanol, and absorbance of eluates determined at 590 nm (bottom left). Cell number was measured by direct counting of viable cells in a hemocytometer (bottom right). (**B**) ITPR1 knockdown and control C2C12 myoblasts were starved with or without mimosine for 24 h and released for 6 h before collection. Cells were permeabilized and stained with PI, prior to FACS analysis. The percentage of cells within each cell cycle phase (G1, S, and G2/M) was determined based on the DNA content. (**C**) Cell lysates from ITPR1 knockdown C2C12 myoblasts were analyzed by immunoblotting with anti-phospho EGFR, anti-Ras (GTP-bound form), anti-phospho ERK1/2, anti-ERK1/2, and anti- phospho CDK2 antibodies, with GAPDH as the loading control. s counted based on DAPI staining.

In our experiments, C2C12 myoblasts with ITPR1 knockdown displayed a higher proliferation rate, compared with control C2C12 myoblasts (Fig. 3A). FACS analysis after PI staining disclosed an increased S phase population in ITPR1 knockdown cells (Fig. 3B). As cell cycle arrest is a prerequisite for myogenic differentiation [31,32], these results suggest that ITPR1 plays a critical role in cell cycle exit for myogenic differentiation.

Multiple studies suggest an important role for the Ras-ERK1/2 pathway in the development, maintenance, and pathology of mammalian skeletal muscle. ERK activity promotes the proliferation of myoblasts and the terminal differentiation of myotubes, but ERK is inhibitory for muscle specific gene expression at early stage of differentiation [33, 34]. EGFR downregulation is essential for the induction of myoblast differentiation at least in part by down-regulating the ERK pathway [35]. While EGFR-Ras-ERK signaling is necessary for cell cycle progression, termination of this pathway is required for the initiation of myogenic differentiation. Here we found that ITPR1 knockdown C2C12 myoblasts exhibited higher EGFR phosphorylation, GTP-bound Ras activation and ERK phosphorylation. Moreover, ITPR1 depletion induced CDK2 phosphorylation, leading to S phase progression in C2C12 myoblasts (Fig. 3C).

**Figure 4:**
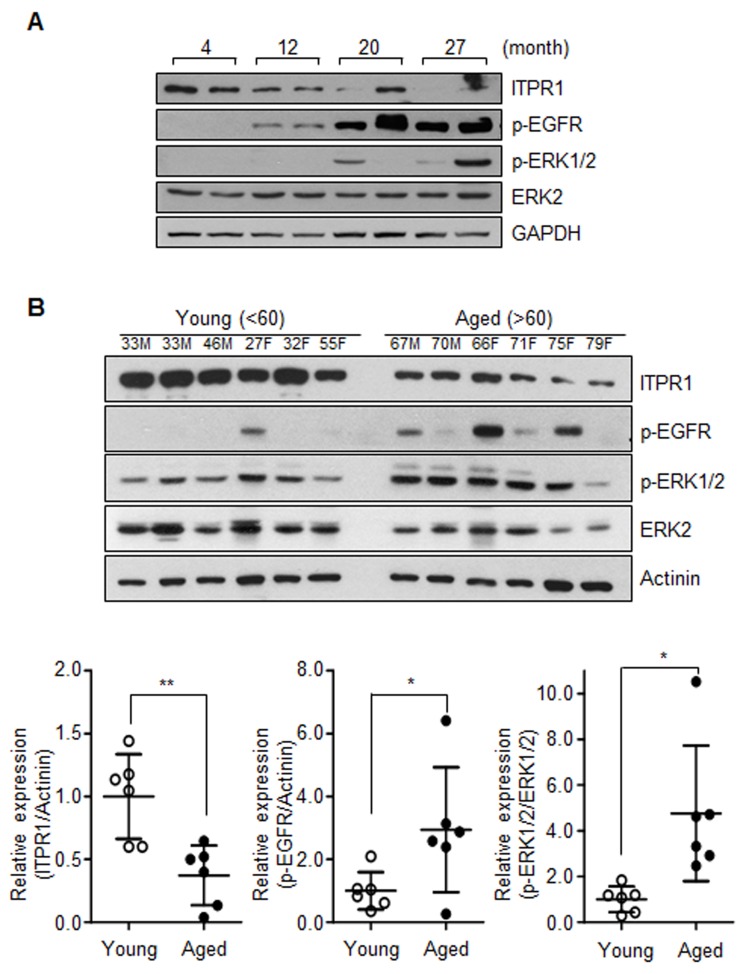
Activation of the EGFR-Ras-ERK pathway in aged muscle (**A**) Immunoblot analysis of quadriceps tissue extracts from 4, 12, 20, 27 months mice with anti-ITPR1, anti-phospho EGFR, anti-phospho ERK1/2, and anti-ERK2 antibodies. GAPDH was used as the loading control. *n* = 2 for each group. (**B**) Immunoblot analysis of human quadriceps (vastus lateralis) derived from young and aged individuals with anti-ITPR1, anti-phospho EGFR, anti-phospho ERK1/2, and anti-ERK2 antibodies. Actinin was used as the loading control. Young subjects represented individuals less than 60 years of age while aged subjects were greater than 60 years of age. *n* = 6 for each group.

We further investigated whether EGFR-Ras-ERK signaling is activated in aged skeletal muscle with decreased ITPR1 expression. Notably, the age-related ITPR1 decline in mice and human skeletal muscles was correlated with increased activation of EGFR-Ras-ERK signaling (Fig. 4A, B).

### ITPR1 is required for normal muscle regeneration

**Figure 5:**
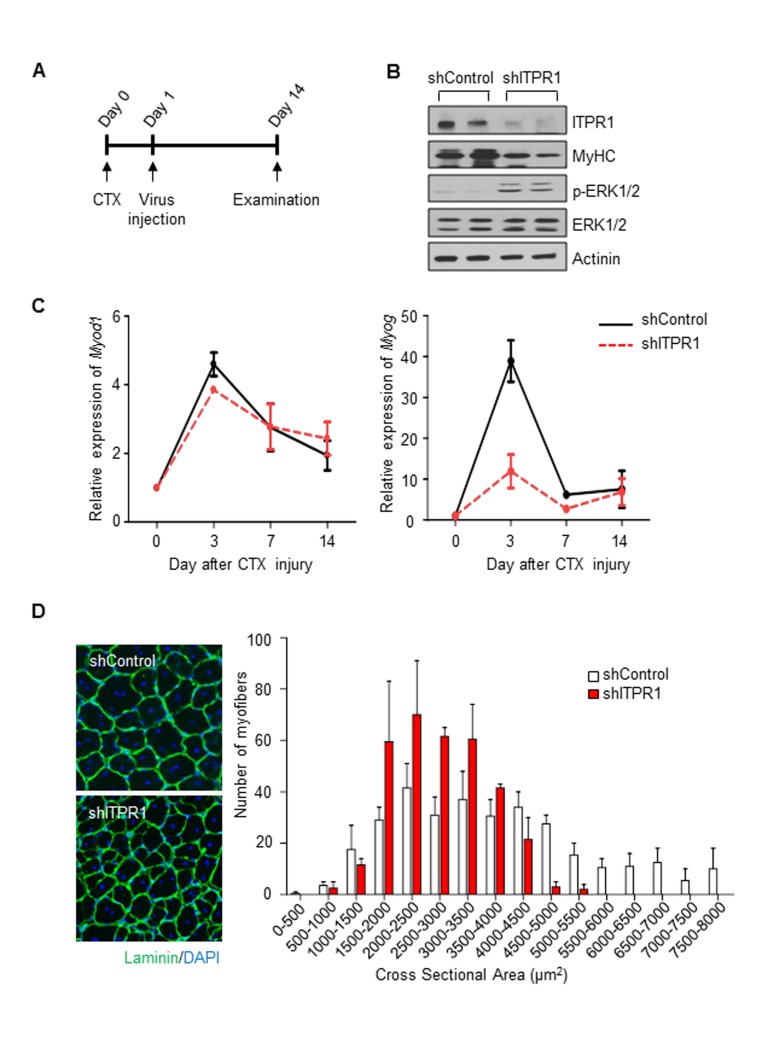
Intramuscular adenovirus-mediated ITPR1 silencing delays muscle regeneration (**A**) Scheme of the *in vivo* experimental procedure. TA muscles of C57BL/6 mice were injected with 50 μl of 20 μM CTX using an insulin syringe at day 0. The next day, injured TA muscle tissues were injected with adenovirus encoding shITPR1-RFP or scrambled shRNA-RFP (1.4×10^9^ particles) using an insulin syringe. At the indicated days, muscle tissues were harvested for biochemical and histological analyses. (**B**) Immunoblot analysis of ITPR1 knockdown and control mouse TA muscle lysates with anti-ITPR1, anti-MyHC, anti-phospho ERK1/2 and anti-ERK1/2 antibodies, with actinin as the loading control. (**C**) qRT-PCR analysis of the time-courses of *Myod1* and *Myog* mRNA expression in ITPR1-silenced and control TA muscle samples subjected to CTX injury, relative to *36b4*. *n* = 3 for each group. (**D**) Representative immunohistochemistry images of Laminin (green) and DAPI (blue) staining of ITPR1-silenced and control TA muscle fibers on day 14 after CTX injury. Cross-sectional areas of muscle fiber were measured using NIS-Elements Microscope Imaging software (Nikon) and fiber size distributions are presented as means ± S.D. (*n* = 3).

Recovery of skeletal muscle after injury depends on myogenic progenitor cell activation and differentiation, which are reduced in sarcopenia. To ascertain whether ITPR1 silencing disrupts muscle regeneration in vivo, adenovirus encoding shITPR1-RFP or scrambled shRNA-RFP was injected into contralateral TA muscle of 6 week-old C57BL/6 male mice the day after CTX injury (Fig. 5A). At 6 days after CTX injury, decreased ITPR1 expression was evident in shITPR1-infected muscle (Fig. 5B). ITPR1 knockdown led to reduced MyHC content, indicating defective muscle differentiation and enhanced ERK activation (Fig. 5B). These phenotypes were accompanied by lower expression of the myogenic transcription factor Myog (Fig. 5C). As myogenin participates in myocyte fusion during the late stage of myoblast differentiation [31], we propose that ITPR1 plays a role during this period. Another myogenic transcription factor Myod1, participating in myoblast proliferation rather than differentiation [2, 36], was not differentially expressed in ITPR1 knockdown muscle. At 14 days after CTX injury, we performed immunohistochemical analysis to measure the cross-sectional area (CSA) of regenerating fibers. As shown in Fig. 5D, the newly formed myofibers with central nuclei had smaller diameters in ITPR1 knockdown muscle (1,500–3,000 μm2), relative to control muscle (2,500–4,000 μm2). These findings clearly indicate that ITPR1 expression is necessary for proper muscle regeneration.

### An ERK inhibitor restores impaired muscle regeneration in aged mice

**Figure 6:**
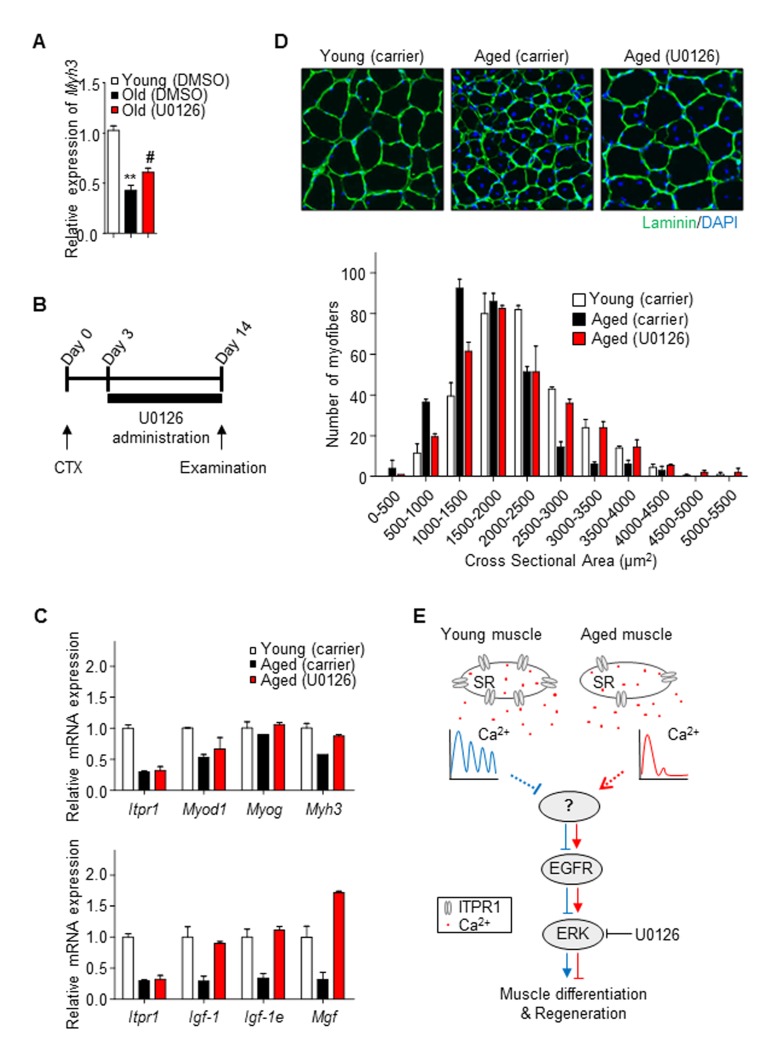
Restoration of aged muscle by ERK inhibition (**A**) Old primary myoblasts were induced to differentiate in the presence or absence of U0126 (5 μM) for 2 days. *Myh3* mRNA levels was measured relative to *36b4* using qRT-PCR. (**B**) Scheme of the *in vivo* experimental procedure. Mouse TA muscles were injected with 50 μl of 20 μM CTX using an insulin syringe at day 0. Three days after CTX injury, U0126 was injected intraperitoneally (10 mg/kg) daily. Control young and aged mice were injected with carrier solution for 14 days. (**C**) qRT-PCR analysis of mRNA expression of *Itpr1*, myogenesis regulatory genes (*Myod1, Myog, Myh3*) and muscle hypertrophic regulatory genes (*Igf-1, Igf-1e, Mgf*), relative to *36b4*, in TA muscles of young, U0126- and carrier- injected aged mice. *n* = 3 for each group. (**D**) Representative immunohistochemistry images of Laminin (green) and DAPI (blue) staining of TA muscle fibers from U0126- and carrier- injected mice 14 days after CTX injury. Cross-sectional areas of muscle fibers were measured using NIS-Elements Microscope Imaging software (Nikon) and fiber size distributions are presented as means ± S.D (*n* = 3 for each group). (**E**) Schematic illustration of our model.

To establish whether ERK activation is responsible for inhibition of myogenesis, the ERK pathway was blocked with a specific inhibitor, U0126, in old primary myoblasts. Treatment with U0126 restored Myh3 mRNA expression (Fig. 6A), suggesting that the ERK pathway is involved in age-associated degeneration of muscle differentiation.

To further evaluate the therapeutic potential of ERK signaling inhibitors for sarcopenia, we examined the effects of U0126 on impaired muscle regeneration in aged mice. U0126 (10 mg/kg mouse) or carrier solution was injected intraperitoneally (i.p.) on a daily basis into 6 and 24 month-old C57BL/6 male mice for 13 days after CTX injury (Fig. 6B). Quantitative real-time PCR data revealed that U0126 induced higher expression of not only myogenic regulatory genes (Fig. 6C, upper) but also those involved in hypertrophy in aged muscle (Fig. 6C, lower). Consistently, muscle CSA measurements revealed that the newly formed myofibers of U0126-treated muscle had significantly larger diameters than those of carrier solution-treated aged TA muscle (Fig. 6D), supporting the potential of ERK inhibitors as new candidate therapeutic agents for sarcopenia.

## DISCUSSION

In this study, we have reported age-related changes in Ca^2+^ homeostasis and consequent effects on muscle physiology. Skeletal muscle is maintained by myogenic progenitors in which asymmetric division processes, including proliferation and differentiation, are tightly regulated in response to physiological and pathological cues. Similar pivotal mechanisms in proliferation and differentiation of muscle progenitor cells were previously demonstrated to be controlled by Foxo3-Notch signaling [37, 38]. While the importance of myogenic transcription factors in regulating myoblast proliferation and differentiation is relatively well established [9, 19], limited information is available on the potential roles of cellular environmental components, such as Ca^2+^, in determining myogenic progenitor fate. Our emerging data support the theory that cellular Ca^2+^ mobilization influences myoblast proliferation and differentiation in an age-dependent manner.

With aging, skeletal muscle shows impaired myogenic potential. Myogenesis is completely Ca^2+^ dependent. Ca^2+^ channels in muscle include RyRs, DHPRs, ITPRs, STIM, ORAI, and TRPCs. Among the various Ca^2+^ channels, RyR1 is modified with aging by oxidation of its cysteine residues, which causes intracellular Ca^2+^ leakage [8]. Significant uncoupling of RyR with DHPR in aged skeletal muscle fiber also results in reduced peak intracellular Ca^2+^ [39]. However, these modifications do not affect myogenic potential. Therefore, we think that alternative mechanisms may be involved in the impaired myogenic potential that occurs with age. Whereas RyR1 is not expressed in undifferentiated myoblast, ITPR1 is expressed at high levels in young myoblasts, but at low levels in old myoblasts. Therefore, we are interested in ITPR1 as a candidate for causing the impairment in myogenesis with age. Here, we have provided new evidence that decreased expression of ITPR1 triggers dysregulation of Ca^2+^ oscillation, which in turn modulate gene expression, resulting in defective myogenesis. Ca^2+^ oscillation is known to modulate gene expression in many tissues, including muscle [18].

Another interesting finding was EGFR-Ras-ERK activation in aged mice hindlimb muscles (Fig. 4A), which was consistently observed in ITPR1-knockdown C2C12 cells (Fig. 3C). The EGFR-Ras-ERK pathway is one of the most important control mechanisms for cell growth and proliferation via regulating molecules involved in cell cycle arrest and progression in mammals [40]. Previously, downregulation of EGFR activity during human myoblast differentiation was shown to be required for normal differentiation [35]. Reportedly, Ras inhibits myogenic differentiation in a manner dependent on loss of MyoD expression [41]. Another group demonstrated that ERK is involved in the pathogenesis of muscle wasting in cancer cachexia and proposed its utility as a therapeutic target [42]. Accordingly, we hypothesized that the EGFR-Ras-ERK pathway may function as an important negative regulator in aged muscle.

Not only myoblast differentiation is required for muscle regeneration, the initial burst of myoblast proliferation is also necessary [43]. Paradoxically, a reduction in ITPR1 expression may induce an initial burst of myoblast proliferation, while inhibiting subsequent differentiation into myotubes. However, in our in vivo experiments, we could not observe effects on the initial burst, because ITPR1-knockdown constructs were injected 1 day after CTX injury (likely after the initial burst had already occurred). In fact, ITPR1 knockdown had no effect on Myod1 levels (myoblast proliferation), but dramatically increased MyoG levels (differentiation) (Figure 5). Therefore, in the present study, we would suggest that the role of ITPR1 is restricted to myogenic differentiation only. Ultimately, future studies using muscle-specific ITPR1-knockout mice may also reveal ITPR1 effects on the initial burst.

Based on our observations and previous reports, we carefully speculate how lowering ITPR1 increases EGFR phosphorylation. The detailed sequence of events is as follows: (1) lowering ITPR1 decreases Ca^2+^ oscillations (Figure 1F and Supplementary Figure S3); (2) Ca^2+^ oscillations induce calcineurin activity, as previously reported [44–46]; and (3) reduced calcineurin activity results in EGFR phosphorylation (Fig. 6E). The latter conclusion is based on the demonstration that calcineurin dephosphorylates EGFR at a phospho-tyrosine residue in vitro [47, 48]. Confirming this IP3R1- Ca^2+^ oscillation-calcineurin-EGFR axis will require further investigation.

Regulation of the EGFR-Ras-ERK pathway may thus enhance the volume and strength of aged skeletal muscles. Surprisingly, intraperitoneal treatment of muscles of aged mice with the ERK inhibitor, U0126, enhanced the expression of skeletal muscle hypertrophic response genes, such as the Igf-1 family, as well as myogenic regulatory genes Myod1, Myog and Myh3, and induced morphology typical of young muscle. Our results strongly support the utility of ITPR1 as a promising new therapeutic target for age-related muscle degeneration, which can be treated using ERK inhibition strategies.

## MATERIALS AND METHODS

### Cell culture and mouse tissue preparation

Mouse C2C12 myoblasts were grown in DMEM supplemented with 10% FBS, 20 mM HEPES, 2 mM L-glutamine, and antibiotics (Life Technologies Corp., Carlsbad, CA, USA) at 37°C in a humidified atmosphere containing 5% CO2. Cells were expanded in growth medium (GM) and differentiated into myotubes in differentiation medium (DM, DMEM with 2% horse serum).

Six or 28 month-old C57BL/6 male mice were purchased from the Laboratory Animal Resource Center in KRIBB. Live animals were euthanized according to the protocols approved by the Animal Care and Use Committee of KRIBB. Mice were sacrificed via cervical dislocation, and the muscles from each animal immediately dissected. Muscle tissues were frozen in liquid nitrogen and stored at −80°C until processing. Human normal vastus lateralis skeletal muscle tissues of young and aged subjects were obtained from Seoul National University Bundang Hospital (SNUBH). This study was approved by the institutional review board of SNUBH (B-1307-212-008).

### Western blotting

Cells were sonicated (1 sec on and 1 sec off at 20% amplitude for 20 sec) in mammalian cell lysis buffer (20 mM HEPES, pH 7.2, 50 mM NaCl, 0.5% Triton X-100, 10% glycerol) containing both protease and phosphatase inhibitors. Mouse skeletal muscle tissues were homogenized in RIPA buffer (50 mM Tris, pH 7.2, 150 mM NaCl, 1% Triton X-100, 1% Na-deoxycholate, 0.1% SDS) containing both protease and phosphatase inhibitors. Lysates were cleared by centrifugation at 14,000 x g for 20 min, and protein amounts in the supernatant measured using the BCA (Pierce Biotechnology Inc., Rockford, IL, USA) assay. The resulting supernatant fractions were subjected to SDS-PAGE followed by western blot. Antibodies against Actinin, ERK1/2, ERK2, Myogenin, MyHC, Troponin C and β-Actin were obtained from Santa Cruz Biotechnology Inc. (Dallas, TX, USA) while the ITPR1 antibody was from Abcam (Cambridge, MA, USA). Antibodies against phospho-CDK2, phospho-EGFR, and phospho-ERK1/2 were purchased from Cell Signaling Technology Inc. (Danvers, MA, USA). The antibody against GAPDH was developed in our laboratory.

### Immunofluorescence

Cells grown on 6-well plates with coverslips were cultured in DM. At the indicated times, cells were fixed with 3.7% paraformaldehyde (PFA) in PBS for 15 min and permeabilized in 0.1% Triton X-100 in PBS for a further 15 min. After blocking in 2% BSA in PBS for 30 min, cells were incubated with anti-MyHC antibody (1:100 dilution) for 16 h at 4°C, and further with secondary FITC-conjugated antibody for 1 h at room temperature. Coverslips were mounted on glass slides with mounting medium containing DAPI and analyzed under a fluorescence microscope.

### RNA isolation and Reverse Transcription-PCR

Isolation of total RNA from skeletal muscle tissues or C2C12 myoblasts was performed using RiboEX reagent (GeneAll Biotechnology Co., South Korea). RNA preparation and cDNA synthesis were performed according to manufacturer's protocols. PCR was conducted with the following primers for mouse: Itpr1 Forward, 5′-TGG CAG AGA TGA TCA GGG AAA-3', Itpr1 Reverse, 5′-GCT CGT TCT GTT CCC CTT CAG-3', Itpr2 Forward, 5′-GCT CAG ATG ATC ACG GAG AAG-3', Itpr2 Reverse, 5′-ATC TCA TTT TGC TCA CTG TCA CCT-3', Itpr3 Forward, 5′-TCA TTG TAC TGG TCC GAG TCA AGA-3', Itpr3 Reverse, 5′-GCG GGA ACC AGT CCA GGT-3', Actb Forward, 5′-CAC TAT TGG CAA CGA GCG GT-3' and Actb Reverse, 5′-CTT CAT GGT GCT AGG AGC CA-3'.

### Quantitative real-time PCR (qRT-PCR)

Differential expression of selected genes was examined using qRT-PCR with the SYBR Green detection system on an ABI 7300 real-time PCR machine (Applied Biosystems, Foster City, CA, USA). Amplification reactions were performed as follows: one cycle at 95°C for 10 min, followed by 40 cycles of 95°C for 15 sec and 60°C for 1 min. The threshold cycle (Ct) is defined as the fractional cycle number at which the fluorescence passes the fixed threshold. Data were normalized to the abundance of 36b4 mRNA in each reaction. The primer sequences are listed in Supplementary Table S1.

### RNA interference and transfection

For siRNA transfection, C2C12 myoblasts were transfected with 50 pmol siRNA using Lipofectamine RNAiMax (Life Technologies Corp.) according to the manufacturer's protocol. All siRNA sequences were purchased from Bioneer Corp. in South Korea. To establish a stable ITPR1 knockdown cell line, small hairpin RNA (shRNA) against mouse ITPR1 (clone ID NM_010585.2-4576s1c1) in pLKO.1-puro lentiviral vector was purchased from Sigma-Aldrich (St. Louis, MO, USA). 293T cells containing shRNA lentiviral particles were generated by transient transfection with pLP1, pLP2, pVSV-G (Life Technologies Corp.) and shRNA lentiviral or pLKO.1-scrambled (control) vector (SHC002V; Sigma-Aldrich) using Lipofectamine (Life Technologies Corp.), in keeping with the manufacturer's protocol. Forty-eight hours after transfection, supernatant fractions containing lentiviral particles were collected and used to infect C2C12 myoblasts in the presence of 4 μg/ml polybrene. Infected cells were selected by incubation with 2 μg/ml puromycin for 2 weeks. For ectopic expression of ITPR1, Myc-tagged rat ITPR1 plasmid was transfected into C2C12 ITPR1 knockdown cells using Lipofectamine. To select stably transfected cells, 1 mg/ml G418 was added to the growth medium for 3 weeks.

### Cell proliferation assay

C2C12 myoblasts (100 cells) were seeded into 6-well plates in triplicate and incubated at 37°C for 7 days. Cells were fixed with 3.7% PFA for 15 min, stained with a 0.5% crystal violet solution for 2 h, rinsed with distilled water, and air-dried. Cell-bound crystal violet was dissolved with 1:1 solution of 100 mM sodium citrate in ethanol, and the absorbance measured at 590 nm using a UV-VIS spectrophotometer. Dye-stained cells were trypsinized for detaching from the plate and counted using a hemocytometer.

### Cell cycle analysis

Cell cycle progression was assayed as described previously [40]. Briefly, cultured cells were treated with mimosine, an inhibitor of DNA replication, for 24 h, replaced in normal medium (DMEM with 10% FBS) for 6 h, and harvested via trypsinization. An aliquot of cells (∼1×106) was washed with PBS and fixed with 3.7% PFA. Prior to flow cytometry, cells were suspended in PBS containing 50 μg/ml propidium iodide (PI) and 10 μg/ml DNase-free RNase (Sigma-Aldrich). Flow cytometry was performed using a fluorescence-activated cell sorter (FACSCalibur; BD Biosciences, San Jose, CA, USA) containing CELLquest software. Phases of the cell cycle (G1, S and G2/M) were analyzed using the ModFit LT program (Verity Software House Inc., Topsham, ME, USA).

### Ras pull-down activity assay

Activated Ras (GTP-Ras) pull-down assays were performed according to the manufacturer's protocol (Stressgen Biotechnologies Corp., San Diego, CA, USA). Briefly, cell lysates were incubated with Ras-binding domain (RBD) of Raf-1 agarose beads at 4°C for 1 h. After washing, activated Ras bound to Raf-1 RBD agarose beads was released by the addition of 2x SDS sample buffer and monitored via immunoblotting with a monoclonal pan-Ras antibody.

### Calcium imaging

Intracellular Ca^2+^ imaging was performed as described previously [49]. Imaging was carried out using an inverted confocal microscope (LSM 510 META, Carl Zeiss, Oberkochen, Germany) with a 40x objective. To monitor cytosolic Ca^2+^ levels, C2C12 myoblasts or primary myoblasts were seeded within a 6 channel μ-slide flow chamber (Ibidi GmbH, Martinsried, Germany) at low density. The next day, cells were loaded with 2 μM Fluo-4 acetoxymethylester (Fluo-4 AM, Life Technologies Corp.) in a physiological salt solution (PSS: 150 mM NaCl, 4 mM KCl, 2 mM CaCl2, 1 mM MgCl2, 5 mM glucose, 5 mM HEPES) for 30 min at 37°C. Cells were further washed with PSS for 30 min at 37°C to allow complete de-esterification of the dye. Fluo-4 was excited with a 488 nm laser line and fluorescence acquired at wavelengths of 505-530 nm. Cells were treated with ATP (Sigma-Aldrich) for intracellular Ca^2+^ perturbation.

### Skeletal muscle injury and Adenovirus delivery

Six week-old male C57BL/6 mice were obtained from the Laboratory Animal Resource Center in KRIBB. Prior to experiments, mice were acclimatized to a 12 h light/dark cycle at 22 ± 2°C for 2 weeks with access to unlimited food and water under specific pathogen-free conditions. To induce skeletal muscle injury, mice were anesthetized with 1–3% isoflurane/O2 and injected with 50 μl of 20 μM cardiotoxin (CTX, Sigma-Aldrich) solution into TA muscle using an insulin syringe. The needle was inserted parallel to the longitudinal muscle fiber until the tip reached the tendon near the knee and then slowly withdrawn while injecting the CTX solution in its path. The following day, 1.4×109 particles of adenovirus encoding shITPR1-RFP or scrambled shRNA-RFP (Vectorbiolabs, Malvern, PA, USA) were injected into the TA muscle using an insulin syringe for delivery of ITPR1 knockdown virus.

### U0126 injection into mice

U0126 was prepared in DMSO as a stock solution of 10 μM, and the amount of drug to be injected into mice was diluted with carrier solution (40% DMSO in PBS). In total, 200 μL U0126 (10 mg/kg mouse body weight) was injected intraperitoneally (i.p.) daily into each mouse. Control mice were injected with carrier solution.

### Histological analysis of muscle tissue

Mouse TA muscle tissues were embedded with an optimal cutting temperature (OCT) compound (Sakura Corp., Osaka, Japan) and immediately frozen in dry ice-cooled isopentane. Muscles in OCT were cut into 10 μm thick cryosections with a cryostat (Thermo Electronic Corp., Waltham, MA, USA) maintained at −20°C. Sections were stained with hematoxylin and eosin (H&E) and examined using light microscopy. For immunohistochemistry, serial sections were air-dried for 30 min and fixed in 4% PFA for a further 30 min. After washing in PBS, sections were blocked in 5% BSA and incubated with primary rabbit anti-Laminin antibody (Sigma-Aldrich) in 5% BSA at 4°C overnight, followed by FITC-conjugated rabbit-IgG (Life Technologies Corp.) for 30 min. Sections were further rinsed in PBS and cover-slipped. Slides were visualized with a Nikon ECLIPSE Ti-U inverted microscope and Nikon DS-Ri2 camera using NIS-Elements software.

### Statistical analysis

Results are expressed as means ± S.D. Student's unpaired t-test was applied to compare quantitative data. Data were considered statistically significant at p-values < 0.05 (*p < 0.05, **p < 0.01, ***p < 0.001).

## SUPPLEMENTARY MATERIAL TABLE AND FIGURES



## Abbreviations

CTX: Cardiotoxin
DM: differentiation medium
GM: growth medium
ITPR1: inositol 1,4,5 triphosphate receptor type 1
MyHC: myosin heavy chain
TA: tibialis anterior
