# Supplementary material for: Age-associated repression of type 1 inositol 1, 4, 5-triphosphate receptor impairs muscle regeneration
Source: Aging (Albany NY). 2016 Sep 21;8(9):2062–76. doi: 10.18632/aging.101039 (PMC5076452; doi:10.18632/aging.101039)
Supplement: Supplementary file 1 [file aging-08-2062-s001.pdf]

SUPPLEMENTARY MATERIAL

Table S1. Primer Sequences for quantitative RT-PCR.

GENE	FORWARD	REVERSE
(m) *Itpr1*	5'-CGT TTT GAG TTT GAA GGC GTT T-3'	5'-CAT CTT GCG CCA ATT CCC G-3'
(m) *Itpr2*	5'-CCT CGC CTA CCA CAT CAC C-3'	5'-TCA CCA CTC TCA CTA TGT CGT-3'
(m) *Itpr3*	5'-GGG CGC AGA ACA ACG AGA T-3'	5'-GAA GTT TTG CAG GTC ACG GTT-3'
(m) *Myh3*	5'-AAAAGGCCATCACTGACGC-3'	5'-CAGCTCTCTGATCCGTGTCTC-3'
(m) *Myog*	5'-CTACAGGCCTTGCTCAGCTC-3'	5'-ACGATGGACGTAAGGGAGTG-3'
(m) *Myod1*	5'-CCACTCCGGGACATAGACTTG-3'	5'-AAAAGCGCAGGTCTGGTGAG-3'
(m) *36b4*	5'-AGATTCGGGATATGCTGTTGG-3'	5'-AAAGCCTGGAAGAAGGAGGTC-3'
(m) *Stim1*	5'-GGC GTG GAA ATC ATC AGA AGT-3'	5'-TCA GTA CAG TCC CTG TCA TGG-3'
(m) *Orai1*	5'-GAT CGG CCA GAG TTA CTC CG-3'	5'-TGG GTA GTC ATG GTC TGT GTC -3'
(m) *Tpcn1*	5'-TCC AAG GCC TTC CAG TAT TTC-3'	5'-CTC CAC CAG GAT CCA GAC AC-3'
(m) *Tpcn2*	5'-CAC GAC TGA TGA ACA CAC TGA-3'	5'-CCA GGA GGC ACG ATG ACA C-3'
(m) *Cacna1*	5'-TCA GCA TCG TGG AAT GGA AAC-3'	5'-GTT CAG AGT GTT GTT GTC ATC CT-3'
(m) *Ryr1*	5'-CAG TTT TTG CGG ACG GAT GAT-3'	5'-CAC CGG CCT CCA CAG TAT TG-3'
(m) *Igf1*	5'-CCA CAC TGA CAT GCC CAA GA-3'	5'-CCT GCA CTT CCT CTA CTT GTG TTC-3'
(m) *Igf1e*	5'-TTC AGT TCG TGT GTG GAC CGA-3'	5'-TCC ACA ATG CCT GTC TGA GGT G-3'
(m) *Mgf*	5'-TTC AGT TCG TGT GTG GAC CG-3'	5'-TGT TTG TCG ATA GGG ACG G-3'

A

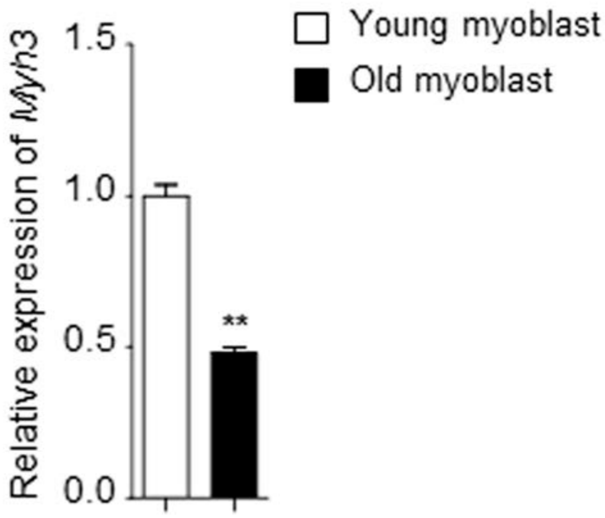

Figure S1. Myotube formation decreases in old myoblast. (**A**) Young and old myoblasts differentiated into myotube and the cells were analyzed mRNA levels of *Myh3* for transcriptional differentiation marker relative to *36B4* using quantitative RT-PCR.

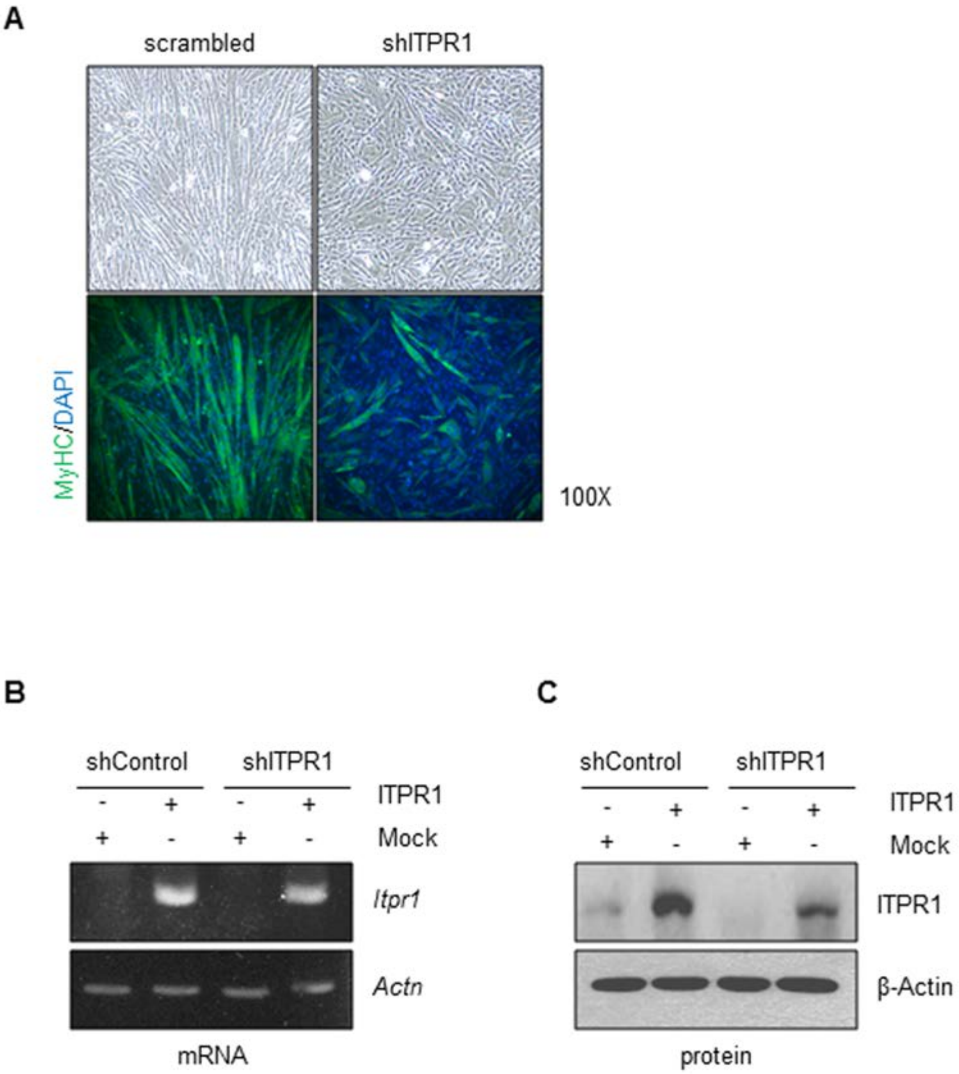

Figure S2. ITPR1 knockdown decrease myotube formation. (**A**) C2C12 ITPR1 knockdown or control C2C12 myoblasts were induced to differentiate for 5 days and stained with MyHC (green) antibody and DAPI (blue). (**B**-**C**) Myc-tagged rat ITPR1 plasmid DNA transfected into C2C12 ITPR1 knockdown and control cells. Exogenous ITPR1 DNA alter the ITPR1 mRNA (**B**) and protein (**C**) expression in ITPR1 knockdown and control cells. β-Actin was used as a loading control.

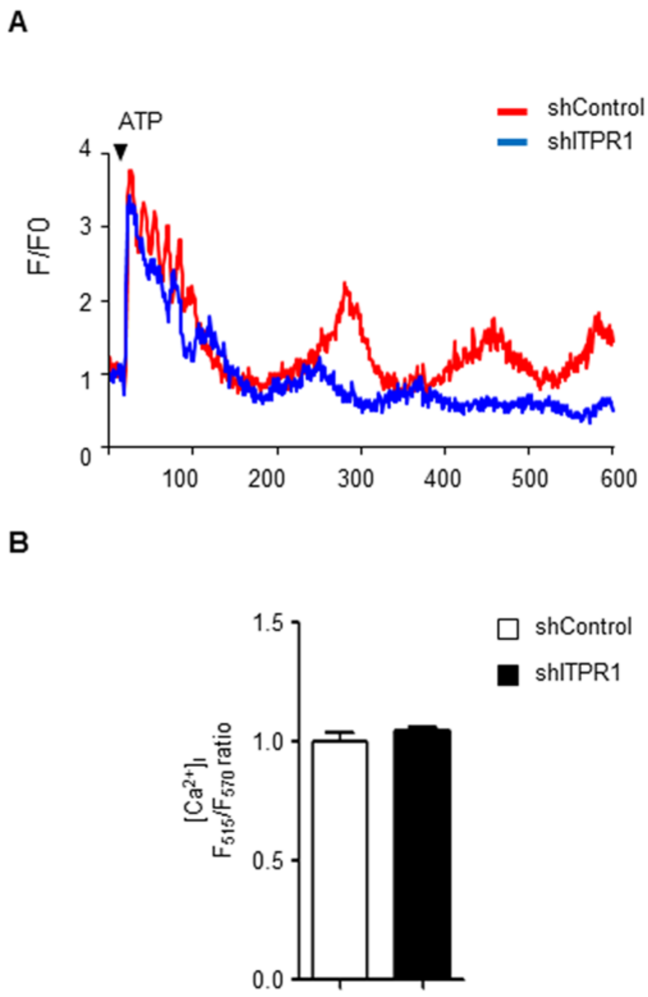

Figure S3. Intracellular Ca^{2+} signaling in ITPR1 knockdown C2C12 cells. (**A**) Ca^{2+} oscillations induced by ATP. ITPR1 knockdown and control C2C12 cells were pre-incubated with 2 μM Fluo-4 AM, Ca^{2+} indicator, for 30 minutes and washed with PSS buffer prior to drug treatment. Fluorescence images were captured by exciting at 488 nm and collecting emissions at 505-530 nm using a time lapse mode of the confocal microscope before and after ATP (10 μM) treatment for 10 minutes. The red line indicates control cells and the blue line indicates ITPR1 knockdown cells. (**B**) Cytosolic resting Ca^{2+} concentration. ITPR1 knockdown and control C2C12 cells were pre-incubated with 2 μM Fluo-4 AM and 2 μM Fura-red AM, Ca^{2+} indicator, for 30 minutes and washed with PSS buffer. Fluorescence images were captured by exciting at 488 nm and collecting emissions at 515 nm (Fluo-4) and 570 nm (Fura-red), respectively in ITPR1 knockdown and control C2C12 cells. Graph indicate that mean fluorescence ratio (Fluo-4/Fura- red : F_{515}/F_{570}).
